# Abnormal Asymmetry of Brain Connectivity in Schizophrenia

**DOI:** 10.3389/fnhum.2014.01010

**Published:** 2014-12-22

**Authors:** Michele Ribolsi, Zafiris J. Daskalakis, Alberto Siracusano, Giacomo Koch

**Affiliations:** ^1^Dipartimento di Medicina dei Sistemi, Clinica Psichiatrica, Università di Roma Tor Vergata, Rome, Italy; ^2^Laboratorio di Neurologia Clinica e Comportamentale, Fondazione Santa Lucia IRCCS, Rome, Italy; ^3^Temerty Centre for Therapeutic Brain Intervention, Centre for Addiction and Mental Health, University of Toronto, Toronto, ON, Canada

**Keywords:** schizophrenia, connectivity, asymmetry, interhemispheric, diffusion tensor imaging, fMRI

## Abstract

Recently, a growing body of data has revealed that beyond a dysfunction of connectivity among different brain areas in schizophrenia patients (SCZ), there is also an abnormal asymmetry of functional connectivity compared with healthy subjects. The loss of the cerebral torque and the abnormalities of gyrification, with an increased or more complex cortical folding in the right hemisphere may provide an anatomical basis for such aberrant connectivity in SCZ. Furthermore, diffusion tensor imaging studies have shown a significant reduction of leftward asymmetry in some key white-matter tracts in SCZ. In this paper, we review the studies that investigated both structural brain asymmetry and asymmetry of functional connectivity in healthy subjects and SCZ. From an analysis of the existing literature on this topic, we can hypothesize an overall generally attenuated asymmetry of functional connectivity in SCZ compared to healthy controls. Such attenuated asymmetry increases with the duration of the disease and correlates with psychotic symptoms. Finally, we hypothesize that structural deficits across the corpus callosum may contribute to the abnormal asymmetry of intra-hemispheric connectivity in schizophrenia.

## Introduction

Left–right asymmetries of brain and behavior are known to be widespread among both vertebrates and invertebrates, and can arise through a number of genetic, epigenetic, or neural mechanisms (Corballis, 2014). In human beings, such brain asymmetry is widely associated with complementary functions, with the left-hemisphere regarded as being dominant for language and handedness (Corballis, 2014), and the right hemisphere as being dominant for some non-verbal functions, such as spatial attention (Cai et al., 2013) and the processing of faces (Dundas et al., 2014). The healthy brain exhibits both structural (e.g., in gray matter volume, in cytoarchitecture and dendritic arborization, or in white-matter integrity) and functional asymmetries (Rentería, 2012). On the contrary, a reduction of this phenomenon has been reported in schizophrenia, mostly as a consequence for the failure of the left-hemisphere dominance (Mitchell and Crow, 2005). Furthermore, a growing body of evidence has revealed that in schizophrenia, such a reduced brain asymmetry is not only structural (Friston, 2002; Koch et al., 2008; Ribolsi et al., 2009; Baker et al., 2014; Zhang et al., 2014) but also functional, as there is an abnormal asymmetry of functional connections compared to healthy subjects (Jalili et al., 2010). Starting with a brief overview of the structural and functional asymmetry in healthy brains, in this manuscript, we will focus our attention on the recent works that investigated the asymmetry of functional connectivity in schizophrenia, suggesting that this aspect may represent a neurophysiological feature that is unique to this disorder. Moreover, we will discuss the possibility that an impaired inter-hemispheric connectivity may influence this phenomenon. Finally, the hypothetical relationship between abnormal functional connectivity and psychotic symptomatology will be also taken into consideration.

## Brain Asymmetry of Connectivity in Healthy Subjects

### Structural brain asymmetry in healthy subjects

#### Gray matter

The brain exhibits macroscopic asymmetries (e.g., in volume, fissurization), microscopic asymmetries (e.g., in cytoarchitecture and dendritic arborization), and neurochemical asymmetries, such as dopaminergic and noradrenergic sensitivity (Toga and Thompson, 2003). It has been observed, initially from autopsy and later from imaging, that the frontal right-hemisphere protrudes more anteriorly and is often wider than the left, and the left occipital lobe extends beyond and is often wider than the right in many individuals (see figure of the cerebral torque from Mitchell and Crow, 2005). This phenomenon is refered to as the so-called “Yakovlevian torque” (LeMay, 1976; Toga and Thompson, 2003). This structural morphology reflects differences of gray matter volume (Weinberger et al., 1982; Takao et al., 2013) and cortical thickness (Luders et al., 2006) in frontal (greater in the right), middle (greater in the left planum temporale) and occipital regions (greater in the left). Furthermore, focal asymmetries, which are more easily interpretable with regards to their relationships with brain function, were also demonstrated. Interstingly, the leftward asymmetry of the planum temporale, an auditory cortex region at the back of the superior temporal surface that is involved in phonetic processing (Jäncke et al., 2002) is considered as an anatomical marker of left hemispheric functional specialization for language (Geschwind and Levitsky, 1968; Shapleske et al., 1999). Notably, in this article, we use the expression leftward/rightward asymmetry to refer to the distribution of asymmetry toward the left or the right hemisphere.

#### White matter

Diffusion tensor imaging (DTI) is one of the most widely used magnetic resonance (MR) imaging techniques for assessing brain tissue integrity, and white matter in particular. It is sensitive to the random thermal motions of water and is useful for probing white-matter microstructure and connectivity *in vivo*. Through this technique, white-matter asymmetry has been revealed in several studies in healthy adults (Cao et al., 2003), in the developing human brain (Beaulieu et al., 2005; Snook et al., 2005; Bonekamp et al., 2007), and in a variety of neuropsychological disorders (Fletcher et al., 2010; Kubicki et al., 2011). Leftward asymmetry of the anterior cingulum and of the arcuate fasciculus (Takao et al., 2013), the cortico-spinal tract (Thiebaut de Schotten et al., 2011), and the posterior limb of the internal capsule (Westerhausen et al., 2007) are among the most consistent white-matter asymmetries in healthy conditions. A selection of studies on white-matter asymmetry in healthy subjects and of their main findings is provided in Table 1.

**Table 1:** **Studies on white-matter asymmetry in healthy subjects and schizophrenia patients**.

Study (year, sample size, and methods)	Brain areas	Asymmetry	Main strengths of the study
**STUDIES ON HEALTHY SUBJECTS**			
Cao et al. (2003) 15 healthy subjects DTI (1.5 and 3 T)	Whole brain	Relative anisotropy of water diffusion in the subinsular white-matter greater on the left	Implication for hemispheric lateralization in language and speech
Takao et al. (2013) 857 healthy subjects DTI (3 T)	Whole brain	Large leftward asymmetry in the anterior cingulum and in the superior longitudinal fasciculus	Sample size; high technology
Beaulieu et al. (2005) 32 healthy children DTI (1.5 T)	Whole brain	Left temporo-parietal fractional anysotropy (FA) correlates with reading ability	Focus on developing brain and reading abilities
Snook et al. (2005) 32 healthy children vs. 28 healthy adults DTI (1.5 T)	Whole brain	Leftward FA in the anterior limb of the internal capsule and centrum semiovale in children; leftward FA in the centrum semiovale and rightward FA asymmetry in the globus pallidus in healthy adults	Comparison between children and adults
Bonekamp et al. (2007) 40 children DTI (1.5 T)	Whole brain	Leftward asymmetry of FA in centrum semiovale	Comparison between children and adults
Thiebaut de Schotten et al. (2011) 20 healthy males and 20 healthy females DTI (1.5 T)	Whole brain	Leftward asymmetry in the cortico-spinal tract and the long segment of the arcuate fasciculus	Greater left lateralization of the fronto-temporal segment of the arcuate fasciculus in males
Westerhausen et al. (2007) 30 left and 30 right-handed healthy subjects DTI (1.5 T)	Cortico-spinal tract	Higher FA in the left hemisphere	Association with handedness
**STUDIES ON SCHIZOPHRENIA PATIENTS**			
Miyata et al. (2012) 26 SCZ DTI (1.5 T)	Whole brain	Similar patterns of overall FA asymmetries in healthy subjetcs and SCZ	The rightward-shift of FA in the external capsule correlates with negative symptom severity
Kubicki et al. (2002) 15 SCZ DTI (1.5 T)	Uncinate fasciculus	Lack of normal left-greater-than-right asymmetry	Focus on a pivotal brain area
Kitis et al. (2012) 11 deficit SCZ 18 non-deficit SCZ DTI (1.5 T)	Uncinate fasciculus	FA reduced in the left uncinate fasciculus of deficit SCZ	Comparison between deficit and non-deficit SCZ
Kunimatsu et al. (2008) 19 SCZ DTI (1.5 T)	Superior occipitofrontal fasciculus	FA reduced in the left occipitofrontal fasciculus	Focus on a specific brain area
Fujiwara et al. (2007) 42 SCZ DTI (3 T)	Cingulum bundle	Left FA accentuated in anterior cingulum bundle	High technology; correlation with positive symptoms
Carletti et al. (2012) 32 ultra high risk of psychosis 15 first-episode SCZ DTI (1.5 T)	Whole brain	Reduced FA values in the left superior corona radiata, left superior occipitofrontal fasciculus	Investigation of high risk subjects
Curčić-Blake et al. (2013) 17 SCZ TBSS	Language network	Reduced fractional anisotropy bilaterally	Significant correlation between the FA decrease in the left arcuate fasciculus and auditory-verbal hallucinations

### Asymmetry of the functional connectivity in healthy subjects

However, beyond structural brain asymmetry, we are now aware of various studies that focus on the asymmetry of the functional connectivity between the two hemispheres (Table 2). Functional magnetic resonance imaging (fMRI) has been used to examine the hemispheric differences in the functional networks. For instance, in a resting-state fMRI study on the hemispheric asymmetry in the cognitive division of anterior cingulate cortex, the connections linking the anterior cingulate cortex to the frontal and parietal lobes are stronger in the right than in the left hemisphere (Yan et al., 2009). Similarly, a comparison between left and right fronto-parietal network by means of EEG during an auditory location oddball paradigm has revealed a rightward hemispheric asymmetry in this network that conforms to the right-hemisphere dominance model in audiospatial perception (Dietz et al., 2014). To this regard, a recent fMRI study has reported two distinct forms of lateralization for the right and the left hemisphere: while the left hemisphere shows a preference to interact more exclusively with itself, particularly for cortical regions involved in language and fine motor coordination, the right-hemisphere cortical regions involved in visuospatial and attentional processing interact in a more integrative fashion with both hemispheres (Gotts et al., 2013). Using the same methods, Saenger et al. (2012) investigated the hemispheric asymmetries of functional connectivity and gray matter volume in the default mode network. For both right-handed and left-handed subjects, a greater leftward functional connectivity was observed in the posterior cingulate gyrus. Interestingly, in this study functional asymmetries are not always reflected or determined by structural asymmetries (Saenger et al., 2012).

**Table 2:** **Studies on asymmetry of functional connectivity on healthy subjects and schizophrenia patients**.

Study (year, sample size, and methods)	Brain areas	Asymmetry	Main strengths of the study
**STUDIES ON HEALTHY SUBJECTS**			
Yan et al. (2009) 49 healthy subjects, resting-state fMRI	Connections linking the anterior cingulate cortex with the frontal and parietal lobes	Greater rightward connectivity	Focus on a brain area involved in cognitive control (cognitive division of anterior cingulate cortex)
Saenger et al. (2012) 171 right-handed subjects and 27 left-handed subjects, resting-state fMRI	Posterior cingulate gyrus	Greater leftward connectivity	Large sample size
Medvedev (2014) 15 healthy subjects. Near-infrared spectroscopy	Connectivity between inferior and middle frontal gyri	Greater rightward connectivity greater flow of information from the right to the left hemisphere	Granger causality as a measure of connectivity
Thatcher et al. (2007) 97 healthy subjects EEG – LORETA	Overall right vs. left hemisphere	Cortical intra-hemispheric coupling stronger in the right hemisphere	Large sample size; investigation of cortical intra-hemispheric coupling
Jalili (2014) 44 healthy subjects resting-state EEG	Overall right vs. left hemisphere	Increased local connectivity in the right hemisphere	Investigation of both global and local efficiency measures
Sun et al. (2014) 26 healthy subjects lower alpha band of EEG during a test of sustained attention	Overall right vs. left hemisphere	Asymmetrical pattern of connectivity (right > left) in fronto-parietal regions	Investigation of asymmetry associated to cognitive functioning
Gotts et al. (2013) 62 healthy males, resting-state fMRI	Overall right vs. left hemisphere	Increased intra-hemispheric connectivity in the left hemisphere; increased inter-hemispheric connectivity in the right hemisphere	Distinct forms of lateralization for the right and the left hemisphere; correlation between degree of lateralization and cognitive functions
Dietz et al. (2014) 12 healthy subjects EEG during an auditory location oddball paradigm	Overall right vs. left hemisphere	Increased right fronto-parietal network	Implications for the explanation of spatial neglect
**STUDIES ON SCHIZOPHRENIA PATIENTS**			
Jalili et al. (2010) 13 SCZ, resting-state EEG	Overall right vs. left hemisphere	Rightward global asymmetry with generally attenuated asymmetry	Correlation with duration disease and negative symptoms
Ke et al. (2010) 16 SCZ with predominantly positive symptoms vs. 16 SCZ with predominantly negative symptoms, resting-state fMRI	Overall right vs. left hemisphere	More positive symptoms → increased leftward asymmetry more negative symptoms → increased rightward asymmetry	Comparison between positive and negative symptoms
Oertel-Knöchel et al. (2013) 24 SZ, resting-state fMRI	Planum temporale	Reduced leftward asymmetry	Focus on a brain area associated with auditory and language processing
Vercammen et al. (2010) 27 SCZ with hallucinations, resting-state fMRI	Coupling between left temporo – parietal junction (TPJ) and bilateral anterior cingulate and the bilateral amygdala	Reduced connectivity of the left TPJ	Correlation with auditory hallucinations
Rotarska-Jagiela et al. (2010) 16 SCZ, resting-state fMRI	Right fronto-parietal network	Lower degree of right-sided laterality with generally attenuated asymmetry	Investigation of the Default Mode Network and correlation with psychopathology
Yan et al. (2012) 33 SCZ resting-state fMRI	Cognitive division of anterior cingulate cortex (ACC-cd)	Abnormal asymmetric connectivity of the ACC-cd with multiple brain areas	Focus on a brain area involved in cognitive control; correlation with Stroop performance
Andreou et al. (2014) 22 first-episode SCZ, resting-state EEG	Overall right vs. left hemisphere	Increased gamma-band left connectivity	Recruitment of first-episode patients: correlation with positive and disorganization symptoms

A recent study used a different approach, by utilizing near-infrared spectroscopy to measure connectivity in each hemisphere between inferior and middle frontal gyri (“unilateral connectivity”), as well as connectivity between homologous structures of both hemispheres (“bilateral connectivity”). There was greater flow of information from the right to the left hemisphere in both left- and right-handed subjects (Medvedev, 2014). In addition, asymmetry of cortical connectivity has been investigated using current source correlations derived from the resting-state EEG. Higher connectivity in the right hemisphere was found in the majority of right-handed subjects (Jalili, 2014). Similarly, Thatcher et al. (2007) have shown that, in general, the right hemisphere exhibited higher intra-hemispheric source correlations than did the left hemisphere. This study confirmed earlier results based on surface EEG coherence, which also found higher right versus left hemispheric coherence (Tucker et al., 1986; Voineskos et al., 2010).

Thus, both electrophysiological and brain imaging studies seem to provide converging evidence that functional connectivity is higher in the right hemisphere (Medvedev, 2014). This pattern has been confirmed during a test of sustained attention (Sun et al., 2014). This right-shifted asymmetric pattern of functional connectivity is not fixed during the life course, but seems to be dependent on both development and aging. For instance, in the neonatal brain, recent studies have revealed a greater structural efficiency in the left hemisphere than in the right, suggesting that the brain regions in the left hemisphere interconnect in better integration and segregation as compared to the right hemisphere (Ratnarajah et al., 2013). Similarly, in a sample of middle age healthy subjects (with a mean age of 53 years), there is a more prominent leftward asymmetry in cortical connectivity, i.e., a larger number of regions demonstrated a higher degree of connectivity in the left hemisphere (Bonilha et al., 2014).

Likewise, cognitive activation studies have consistently demonstrated that prefrontal activation tends to be less lateralized in older adults as compared with younger adults (Cabeza et al., 1997, 2004, Cabeza, 2002; Morcom et al., 2003; Dennis et al., 2007). This phenomenon is known as the HAROLD (hemispheric asymmetry reduction in older adults) effect (Cabeza, 2002), which has also been documented with electroencephalography (Bellis et al., 2000) as well as near-infrared spectroscopy (Herrmann et al., 2006).

## Brain Asymmetry of Connectivity in Schizophrenia

### Structural brain asymmetry in Schizophrenia

#### Gray matter

Several neuroimaging studies have reported a loss of the cerebral torque (greater width of the right frontal and left occipital lobes relative to their contra-lateral counterparts) in patients with schizophrenia compared to normal controls (Crow, 2013). This phenomenon arises out of an abnormality in the genetic control for the development of normal cerebral asymmetry and underlies alterations in language processing (Crow, 1997). In healthy conditions (see figure of the cerebral torque from Mitchell and Crow, 2005), the structure of the cerebral torque produces some effects on the asymmetry of brain connectivity, as the torque drives the transmission within the system in the direction left occipito-temporo-parietal → right occipito-temporo-parietal → right dorso-lateral prefrontal → left dorso-lateral prefrontal (Mitchell and Crow, 2005). Therefore, the core symptoms of schizophrenia may reflect this failure of the left-hemisphere dominance for the faculty of language (DeLisi, 2001; Crow, 2008).

Both structural and functional evidence suggest that schizophrenia is associated with reduced lateralization of language to the left hemisphere, with some studies reporting a reversal of lateralization to the right hemisphere (Gruzelier et al., 1999; Gur and Chin, 1999; Kwon et al., 1999; Aydin et al., 2001; Sommer et al., 2001; Kircher et al., 2002; Sumich et al., 2002). Interestingly, a loss of left-hemisphere dominance and structural anomalies of cerebral asymmetry are related to the progression of the disease (Lam et al., 2012). A second morphological aspect in schizophrenia concerns the abnormal cortical folding (Cachia et al., 2008; Palaniyappan et al., 2014). In the normal population, structural cortical asymmetries, including the lateral temporal cortex, the planum temporale and the superior temporal sulcus are well-documented in adults and children (Toga and Thompson, 2003) and, interestingly, are already present in term infants (Hill et al., 2010). Some studies have revealed an increased or more complex cortical folding in the right hemisphere compared to the left one in both patients and high-risk subjects (Harris et al., 2004, 2007; Narr et al., 2004; Stanfield et al., 2008).

In contrast, regions with significant reductions in gyrification (hypogyria) are predominantly in the left hemisphere (Palaniyappan and Liddle, 2012), confirming the hypothesis of an abnormal brain lateralization. Interestingly, schizophrenia patients (SCZ) with resistant auditory hallucinations show a greater local sulcal index decrease in the superior temporal sulcus bilaterally, and in a peculiar way, also in the left middle frontal sulcus and the diagonal branch of left sylvian fissure (Cachia et al., 2008).

A strict link between gyrification and disruption in neural connectivity in schizophrenia has been hypothesized (White and Hilgetag, 2011). For example, an increased prefrontal gyrification in schizophrenia has been related to increased local short range prefrontal connectivity and reduced long range connectivity (Dauvermann et al., 2012), although this study didn’t investigate the asymmetry of these connections. Therefore, morphological evidence for both the loss of the cerebral torque and the abnormalities of gyrification may provide some anatomical basis for the aberrant connectivity in schizophrenia.

#### White matter

Diffusion tensor imaging investigation has revealed wide white-matter changes in schizophrenia, even at the illness’ onset, as well as before (Kubicki and Shenton, 2014). Interestingly, either patterns of reduced connectivity can be observable across the different stages of the disorder (Wheeler and Voineskos, 2014) in medication-naïve: first-episode patients (Zhang et al., 2014), high-risk subjects (Bloemen et al., 2010; Carletti et al., 2012), and schizotypal subjects (Smallman et al., 2014), suggesting that they may represent a neurobiological trait marker for the schizophrenia spectrum disorders.

With regard to the asymmetry between the two hemispheres, in schizophrenia, there are patterns of overall fractional anysotropy (FA) asymmetries, similar to healthy controls (Miyata et al., 2012). However, SCZ show a significant reduction of leftward asymmetry in some peculiar white-matter tracts, like the uncinate fasciculus (Kubicki et al., 2002; Kitis et al., 2012; Miyata et al., 2012), the inferior occipitofrontal fasciculi (Miyata et al., 2012), the superior occipitofrontal fasciculus (Kunimatsu et al., 2008), and the anterior cingulum bundle (Fujiwara et al., 2007).

Interestingly, the rightward-shift of the FA of the uncinate fasciculus correlates with negative symptom severity (Miyata et al., 2012). Abnormalities in this fasciculus have been more frequently associated with several psychiatric disorders, suggesting that this peculiar alteration of white matter may be involved in the impairment of some cognitive functions, such as episodic memory, language, and social emotional processing (Von Der Heide et al., 2013). Also interesting, different studies showing that left-hemisphere white-matter tracts are more impaired than are right-hemisphere ones are included in a meta-analysis, which investigated a total of 407 patients affected by schizophrenia or related diagnoses (Ellison-Wright and Bullmore, 2009). Two major clusters of altered white-matter integrity were isolated, both of them in the left hemisphere. These clusters are the cerebello-thalamo-cortical circuit and the temporal network interconnecting the frontal lobe, insula, hippocampus/amygdala, and occipital lobe (Ellison-Wright and Bullmore, 2009). Moreover more reduced FA values have been found in high-risk subjects who later developed schizophrenia, as compared to those who did not, in the left temporal white matter at baseline (Bloemen et al., 2010) and in the left superior corona radiata, left superior occipitofrontal fasciculus after the onset of illness (Carletti et al., 2012). A selection of studies and of their main findings on white-matter asymmetry in schizophrenia subjects is provided in Table 1.

On the whole, the DTI findings seem to confirm the hypothesis of a reduced leftward asymmetry. Although most studies revealed decreased FA, some studies found increased FA in specific white-matter tracts in patients with schizophrenia, compared to controls (Alba-Ferrara et al., 2013). These findings of increased FA in schizophrenia are thought to reflect hyperconnectivity (Zhang et al., 2012) or deficient axonal pruning (Alba-Ferrara et al., 2013). Interestingly, some studies have shown a positive correlation between FA and the degree of negative symptoms (Bijanki et al., 2014; Lee et al., 2014). Similarly, in the study conducted by Szeszko et al. (2008), even if lower FA were found in the uncinate fasciculus, inferior fronto-occipital fasciculus, and superior longitudinal fasciculus, higher FA in the left inferior fronto-occipital fasciculus correlated significantly with greater severity of hallucinations and delusions. According to the authors, a possible explanation for the association between positive symptoms and FA values in this tract is that increased FA may render patients more vulnerable to experiencing such symptoms (Szeszko et al., 2008).

However, a growing body of research on the left arcuate fasciculus, which is the key pathway of the language network, has revealed reduced FA in this tract in SCZ with auditory hallucinations (Seok et al., 2007; Catani et al., 2011; de Weijer et al., 2011; Abdul-Rahman et al., 2012; Curčić-Blake et al., 2013). To this regard, a recent meta-analysis of DTI studies has revealed for the first time a significant correlation between the FA decrease in the left arcuate fasciculus and auditory-verbal hallucinations, although a high heterogeneity within the entire dataset of the studies included should be also considered (Geoffroy et al., 2014). These data seem to support our hypothesis of a reduced leftward asymmetry in schizophrenia, and, in addition, that such attenuated left lateralization is related to an increase in positive symptoms.

### Asymmetry of the functional connectivity in Schizophrenia

Apart from the above-described evidence for patterns of reduced asymmetry of gray and white matter, functional connectivity was recently investigated in order to account for possible functional asymmetries. A review of the literature on this theme was conducted in the PubMed database by combining the keywords “connectivity,” “schizophrenia,” and “asymmetry” or “laterality” (Table 2). In a specific study on the asymmetry of functional connectivity, a generally attenuated asymmetry between the two hemispheres was found in chronic SCZ as compared to healthy controls (Jalili et al., 2010). Furthermore, the degree of such abnormality of asymmetry increases with the duration of the disease and correlates with the negative symptoms (Jalili et al., 2010). The result of an attenuated asymmetry of functional connectivity was found also by Rotarska-Jagiela et al. (2010), as patients showed a lower degree of right-sided laterality for the right fronto-parietal network. Interestingly, such decreased hemispheric laterality correlates with increasing symptoms of disorganization.

Other fMRI studies investigated specifically the relationship between abnormal asymmetry of functional connectivity and the psychotic symptomatology (Ke et al., 2010). Notably, patients exhibiting positive symptoms showed significantly increased leftward asymmetry of functional connectivity, as measured by a resting-state fMRI. Interestingly, also first-episode SCZ show an increased left connectivity in the gamma-band compared to controls (Andreou et al., 2014). In contrast, patients with more pronounced negative symptoms exhibited increased rightward asymmetry of functional connectivity (Ke et al., 2010). These findings are consistent with the results of some previous studies, e.g., Gruzelier et al. (1999), who reported that active syndrome SCZ (characterized by positive symptoms) showed left-greater-than-right asymmetry, while patients suffering from deficit syndrome schizophrenia (characterized by more negative symptoms), on the other hand, showed right-greater-than-left asymmetry. A paradigmatic example is the analysis of functional connectivity of the auditory perception network (Rotarska-Jagiela et al., 2009; Gavrilescu et al., 2010; Vercammen et al., 2010).

Brain imaging studies have specifically investigated the lateralization of the planum temporale, finding not only a reduced hemispheric asymmetry (Oertel et al., 2010), which correlates with hallucination severity (Sumich et al., 2005) but also a reduced leftward asymmetry of intrinsic functional connectivity within this area in SCZ and their relatives (Oertel-Knöchel et al., 2013). Therefore, there are complex interactions between the morphological alterations and the resulting dysfunctional connectivity of the network associated with auditory hallucinations. However, despite these preliminary results showing an overall reduction of the asymmetry of functional connectivity, there are still relatively few studies addressing this topic systematically in other cortical networks.

## Inter-Hemispheric Connectivity

### Healthy subjects

The corpus callosum is the largest commissure in the brain and acts as a “bridge” of nerve fibers that connect the two cerebral hemispheres. It plays a crucial role in inter-hemispheric integration and is responsible for normal communication and cooperation between the two hemispheres (Nowicka and Tacikowski, 2011). Several studies have demonstrated a relationship between the corpus callosum size and measures of inter-hemispheric transfer time in split-brain and acallosal patients (Marzi et al., 1991; Forster and Corballis, 1998, 2000; Roser and Corballis, 2002).

A decrease in fiber size and transcallosal connectivity might be related to a reduced need for inter-hemispheric communication due, in part, to increased intrahemispheric connectivity and specialization (Doron and Gazzaniga, 2008). DTI findings on healthy individuals demonstrated an increase in molecular diffusion of the corpus callosum in strongly left-lateralized subjects as compared to moderately left-lateralized, bilateral, or right-lateralized subjects (Westerhausen et al., 2006). Furthermore, patterns of directional symmetry/asymmetry of transcallosal transfer time may be related to the degree of brain lateralization (Nowicka and Tacikowski, 2011).

However, it is still uncertain how the corpus callosum regulates transfer and communication between the two hemispheres. Some studies suggest that the corpus callosum could play an inhibitory role, whereas others say that the corpus callosum serves an excitatory function (Bloom and Hynd, 2005). Anatomical and functional lateralization can be explained by either of the two theories. Lateralization could have originated from an inhibitory function of the corpus callosum by inhibiting the opposing hemisphere, thereby hindering development and allowing for asymmetrical hemisphere development. The excitatory model could have allowed for a unilateral flow of information toward one hemisphere, therefore explaining both the origin of lateralization and the integration between the two parts of the brain (van der Knaap and van der Ham, 2011).

### Schizophrenia patients

Structural deficiencies of the corpus callosum in schizophrenia further point to disrupted inter-hemispheric information transferring (Beaumont and Dimond, 1973). At this moment, there is evidence of reduced functional inter-hemispheric connectivity (Ribolsi et al., 2011) and that it goes along with abnormal brain asymmetry. For example, it has been shown that disrupted inter-hemispheric connectivity is related to diminished lateralization of activation during language processing, and that this finding is specific to schizophrenia (Bleich-Cohen et al., 2012). Additionally, insufficient communication between hemispheres might further affect the developmental process of each individual hemisphere, resulting in the relative lack of asymmetry observed in schizophrenic brains (Innocenti et al., 2003).

Many morphological and neuroimaging schizophrenia studies have detected abnormalities in callosal shape (DeQuardo, 1999; Downhill et al., 2000; Narr et al., 2000; Frumin et al., 2002), size (Arnone et al., 2008; Rotarska-Jagiela et al., 2008), density (Hulshoff Pol et al., 2004; Seok et al., 2007; Wolf et al., 2008), structure (Flynn et al., 2003; Diwadkar et al., 2004; Kubicki et al., 2005), and function (Innocenti et al., 2003). Differences in area and volume of the corpus callosum were greatest in patients whose condition was chronic relative to patients with a first episode, as well as the controls (Collinson et al., 2014).

Recent DTI evidence suggests an inter-hemispheric hypoconnectivity in patients with schizophrenia and their relatives (Whitford et al., 2010; Knöchel et al., 2012), which predicts inter-hemispheric transfer time (Whitford et al., 2011) and psychotic symptoms (Whitford et al., 2010). Using a technique called voxel-mirrored homotopic connectivity (VMHC), a recent study found that the correlation between homologous brain regions was reduced in patients with schizophrenia and schizoaffective disorder. According to the authors, deficits in white-matter connectivity in the corpus callosum could disrupt the synchrony between homotopically connected regions because neural signals are not transmitted with fidelity. Another, not mutually exclusive, explanation is that dysfunctions in local gray matter structure could account for the deficits (Hoptman et al., 2012). Interestingly, the inter-hemispheric resting-state FC of VMHC is also disrupted in unaffected siblings of SCZ (Guo et al., 2014).

## Conclusion

As reported above in this manuscript, several studies on schizophrenia have reported a loss of the developmental brain torsion (the so-called cerebral torque) with resulting failure of the left-hemisphere dominance (Mitchell and Crow, 2005). Interestingly, significant reductions in gyrification (hypogyria) are predominantly in the left hemisphere (Palaniyappan and Liddle, 2012). This morphologic evidence may play a role in the impairment and abnormal asymmetry of neural connectivity (White and Hilgetag, 2011), as well as in confirming the hypothesis of a failure of this hemisphere dominance and an abnormal brain lateralization.

A revision of the more recent functional connectivity studies reveals an overall rightward global asymmetry, both in healthy subjects (Medvedev, 2014) and in SCZ (Jalili et al., 2010). However, the patients show a more generally attenuated asymmetry, which increases with the duration of the disease and correlates with the psychotic symptoms (Jalili et al., 2010; Rotarska-Jagiela et al., 2010). Interestingly, patients exhibiting positive symptoms have significantly increased leftward asymmetry of functional connectivity, while the negative symptom group, in contrast, exhibits increased rightward asymmetry of functional connectivity (Ke et al., 2010). The DTI studies also confirm these data with the SCZ showing a significant reduction of leftward asymmetry in some peculiar white-matter tracts, like the uncinate fasciculus (Kubicki et al., 2002; Kitis et al., 2012; Miyata et al., 2012). Furthermore, different studies have also shown that left-hemisphere white-matter tracts are more impaired than are the right-hemisphere ones (Ellison-Wright and Bullmore, 2009).

An important aspect of brain lateralization in the healthy brain is that the left hemisphere has a greater preference for within-hemisphere interactions, whereas the right hemisphere has interactions that are more strongly bilateral. At a macroscopic scale, this is broadly consistent with proposals that hold that cortical representations are more focal in the left hemisphere and more diffuse in the right hemisphere (Gotts et al., 2013). Therefore, in the healthy subjects, left-hemisphere regions are biased to interact more strongly within the same hemisphere, whereas right-hemisphere regions interact more strongly with both hemispheres. These two different patterns of interaction are associated with left-lateralized functions, such as language and motor abilities, and right-lateralized functions, such as visuospatial attention (Gotts et al., 2013). Therefore, as a consequence of abnormal brain asymmetry and of the failure of the left-hemisphere dominance in schizophrenia, lateralized functions are compromised in schizophrenia. Indeed, different studies have revealed abnormal patterns of connectivity in the left hemisphere in relation to specific psychotic domains (Rotarska-Jagiela et al., 2009; Gavrilescu et al., 2010; Vercammen et al., 2010).

In this paper, we also hypothesize that the abnormal asymmetry of the functional connectivity may be partly due to the well-observed inter-hemispheric communication in schizophrenia. As reported above, there is evidence that reduced functional inter-hemispheric connectivity goes along with abnormal brain asymmetry (Innocenti et al., 2003). Furthermore, deficits in white-matter connectivity in the corpus callosum could disrupt the synchrony between homotopically connected regions because neural signals are not transmitted with fidelity (Hoptman et al., 2012).

One possible hypothesis is that the abnormal asymmetry of connectivity may be related to a dysfunctional inter-hemispheric communication. Indeed, insufficient communication between hemispheres might further affect the developmental process of each individual hemisphere, resulting in the relative lack of asymmetry observed in schizophrenic brains (Innocenti et al., 2003). Future studies should investigate the degree of connectivity of a brain region in relation to the efficiency of the inter-hemispheric connectivity in schizophrenia.

## Conflict of Interest Statement

The authors declare that the research was conducted in the absence of any commercial or financial relationships that could be construed as a potential conflict of interest.
